# Miscellaneous Transactions of the National Dental Association at the Annual Meeting, in Milwaukee, August 6–10, 1901

**Published:** 1901-11-15

**Authors:** 


### MISCELLANEOUS TRANSACTIONS OF THE NATIONAL DENTAL ASSOCIATION AT THE ANNUAL MEETING, IN MILWAUKEE, AUGUST 6-10, 1901.

Dr. J. N. Crouse reported that all proceedings against dentists for infringing the crown patents had ceased except in New York. Several suits were pending in the New York courts which he expected to have tried and to win out this fall.

United States Counsul Jos. H. Worman made the statement in his address that some colleges in this country, by their method of selling diplomas to Germans, were creating a sentiment in Germany against all American degrees of every kind, and the belief was quite prevalent there that degrees could be bought in the United States without trouble and for a very small sum. The association resented the accusation in a most practical way, by appropriation from its treasury a thousand dollars for the prosecution of diploma-selling colleges.

Dr. Bogue, of New York, stated that a committee of American dentists of Paris, France, made an invesgation last year and found that not one of the unprofessional practitioners of Paris who held the American degree was a native-born American.

A hearty vote of thanks was extended to Counsul Worman, the United States Government, and the Governor of Illinois, for the interest they had taken in hunting out the authors of the fraudulent diplomas which had been circulated in Germany.

The committee on necrology reported suitable resolutions on the death of Drs. J. H. McKellops and Wm. H. Morgan, which were adopted by a rising vote.

Dr. Williams Donnelly received a hearty vote of thanks for the valuable work he had done in behalf of the bill providing for dental surgeons in the army, and his effort to secure similar service for the navy was heartily commended.

Dr. Walker, of Section I, reported that during the year sixty-nine original articles on the work of this section had appeared in the various journals.

An effort was made to have the recommendations of President Black in regard to amending the constitution passed upon at this session, but it was found that some matters would need further consideration. It was thought best to defer action to next year.

The ladies in attendance at the meeting were so delightfully entertained by the Milwaukee dentists and their wives, that they asked the association to pass for them a most cordial vote of thanks and appreciation.

The committee having in charge the solicitation of material for the dental section of the National Library and Museum made a favorable report, with suggestions for the future. For lack of time the report could not be discussed, but was adopted.

The committee on oral hygiene in the public schools reported that in New York State several individuals and local societies had taken up the work of visiting the schools and making such examinations as were possible of the conditions of the children's teeth. Dr. Gray, the chairman, said that he had personally taken up the work in his town, and was not only examining the children's teeth, but giving instruction as to the proper care of them.

Dr. Noel of the southern branch of the National Association introduced a resolution that the southern branch be allowed three dollars out of the five dollars each of its members are required to pay for membership, as the two dollars now allowed is insufficient with its limited membership to meet the necessarry expenses.

Dr. Guilford, for Section II, reported as an educational item that two new dental colleges had been formed during the year and one had disbanded. The standard of education was being gradually raised, two schools would this year begin four-year courses, and all the other schools would do so with the session beginning in the fall of 1903. One dental periodical had begun publication during the year just closed, and one had ceased publication. Five text books had been published during the year, and four had issued new editions.

Dr. C. L. Goddard, of the University of California, gave a lantern exhibition illustrating "the incisors and the profile." He showed some very fine slides of a large variety of animals comparing them with the purpose of showing the variations in profile in different animals, each having its own peculiarity and still all having some lines in common. As no paper was read, it is not possible to make a report that would convey the idea of the lecture without illustrations. An interesting discussion followed which can not be reported without the lecture and illustration.

At the same session Dr. G. V. I. Brown gave a lecture, illustrated by lantern pictures, on the treatment of irregularities of the teeth in connection with cleft palate and other diseases of the jaws. This we are also unable to report.

Dr. F. B. Noyes exhibited a few slides showing a valuable work he is doing in showing the structural character of the enamel and dentine of the tooth, and their relations to the methods involved in cavity preparation. The time at Dr. Noyes' disposal was not sufficient for him to show but a few of the many interesting slides he had made, and it was decided that his paper should be held over until next year, when it should be given the privilege of a special hearing, such as its importance demands. It is hoped that Dr. Noyes may be able then to present the results of another year's work. It is the most important work done in recent years.

Dr. William Walker presented a modification of the Kingsley interdental splint, which consists of detachable metal arms for securing the bandages. The arms fit into metallic sockets which are vulcanized into the rubber plate.

The committee to which the President's address was referred, in making its report advised that a committee of three be appointed to devise ways and means for bringing local and district societies into working relation with the National Association.

Drs. H. J. Burkhart, C. N. Johnson and A. R. Melendy were appointed as members of the Foreign Relations Committee, to act with a similar committee appointed by the Faculties Association.

Seventy-three new members were enrolled, the largest number in years.

The contest for place of next meeting was made between Boston, Niagara Falls and Detroit, Niagara Falls winning by one vote.

The weather and entertainment by the dentists of Wisconsin and Milwaukee was all that the most fastidious could wish.

The necessary steps were inaugurated by the St. Louis dentists to have the 1903 meeting held at St. Louis, at the time of the great fair.

The officers elected were published in the September REGISTER, page 482.

